# Cosmetic Benefits of Medium-Depth Chemical Peels for Moderate Acne Lesions and Atrophic Acne Scars: A Single-Arm Clinical Trial in Young Adults

**DOI:** 10.3390/jcm14238598

**Published:** 2025-12-04

**Authors:** Anna Deda, Magdalena Hartman-Petrycka, Marta Gędoś, Martyna Wojciechowska, Dominika Wcisło-Dziadecka

**Affiliations:** 1 Department of Practical Cosmetology and Skin Diagnostics, Faculty of Pharmaceutical Sciences in Sosnowiec, Medical University of Silesia in Katowice, ul. Jedności 8, 41-200 Sosnowiec, Poland; 2 Department of Basic Biomedical Sciences, Faculty of Pharmaceutical Sciences in Sosnowiec, Medical University of Silesia in Katowice, ul. Jedności 8B, 41-200 Sosnowiec, Poland; 3 Student Scientific Society, Department of Practical Cosmetology and Skin Diagnostics, Faculty of Pharmaceutical Sciences in Sosnowiec, Medical University of Silesia in Katowice, ul. Jedności 8, 41-200 Sosnowiec, Poland; marta.gedos@gmail.com (M.G.);

**Keywords:** acne vulgaris, chemical peel, yellow peel, glycolic acid, salicylic acid, retinoic acid, azelaic acid, scar reduction, skin hydration

## Abstract

**Background:** Acne vulgaris is a common inflammatory disorder with significant clinical and psychosocial impacts. Medium-depth chemical peels are increasingly used to manage both active acne lesions and atrophic acne scars. This study aimed to quantitatively assess the clinical effectiveness of a novel multimodal medium-depth chemical peel regimen, yellow peel, in improving acne severity and scar depth, as well as skin hydration and sebum production in patients with mild to moderate facial acne. **Methods:** Twenty patients (17 women and 3 men) aged 20–25 with mild to moderate acne vulgaris underwent two sessions of yellow peel treatment at four-week intervals. The peel protocol combined glycolic acid, salicylic acid, and a multi-acid mask containing retinol, azelaic, phytic, kojic, and salicylic acids. Clinical outcomes were evaluated at baseline, four weeks after the first peel, and two months after the second peel. Assessments included the Investigators Global Assessment (IGA), inflammatory lesion count, 3D scar depth analysis, skin hydration (corneometer), and sebum secretion (sebumeter). **Results:** Yellow peel treatment significantly reduced acne severity, with an 85% decrease in inflammatory lesion counts and over 20% reduction in scar depth. Skin hydration improved significantly across all facial regions, and sebum secretion decreased substantially, enhancing skin barrier function and seboregulation. Statistical analysis confirmed the treatment’s efficacy with sustained improvements two months post-final peel. **Conclusions:** The yellow peel protocol is an effective and well-tolerated adjunct therapy for managing mild to moderate acne and atrophic acne scars. By combining exfoliative, anti-inflammatory, antibacterial, sebostatic, and depigmenting agents, this multimodal approach delivers comprehensive skin improvement. Further large-scale, controlled studies are recommended to confirm long-term safety and efficacy.

## 1. Introduction

Acne vulgaris represents one of the most prevalent and persistent inflammatory disorders affecting the pilosebaceous unit, with its impact observed across a broad demographic spectrum. While it predominantly arises in adolescents, affecting up to 85% of individuals between 12 and 24 years of age, recent epidemiological data demonstrate a growing incidence in older adults as well [1,2,3,4]. There is a significant increase in the incidence of acne vulgaris in adult women. Studies indicate that up to 45% of women aged 21–30 and 12% of women aged 41–50 experience acne [4,5,6]. Although diagnosis rates are higher in females, males typically experience more severe and treatment-resistant manifestations of the condition [3].

Clinically, acne vulgaris presents with a diverse array of lesions, including open and closed comedones, inflammatory papules and pustules, nodulocystic eruptions, post-inflammatory pigmentation, and atrophic or hypertrophic scars. These lesions primarily develop on the face, chest, and back—regions with the highest density of sebaceous glands. The pathogenesis of acne is multifactorial, involving excessive sebaceous gland activity, aberrant follicular keratinization, colonization by *Cutibacterium acnes* (*C. acnes*), and local release of pro-inflammatory mediators. Recent investigations also underscore the importance of hormonal influences—particularly excess androgen—and environmental triggers such as psychological stress, dietary factors, and disturbances in skin and gut microbial communities [6,7,8,9,10,11,12,13,14]. Emerging research increasingly emphasizes the pivotal contribution of cutaneous immune responses and local inflammation to acne pathophysiology, shifting focus away from keratinization defects as primary disease drivers. The role of *C. acnes* in acne development is now understood to be more nuanced; the skin’s microbiome balance and the presence of specific *C. acnes* phylotypes, rather than the organism’s absolute abundance, are central to disease modulation [15,16]. Notably, loss of strain diversity and dysbiosis may precipitate inflammation and lesion formation. In addition, other microorganisms, such as *Staphylococcus epidermidis*, contribute to acne pathogenesis by modulating the growth and activity of *C. acnes*. The delicate interplay between these bacteria maintains a functional equilibrium, and its disruption has been implicated in the initiation of acne outbreaks [8,9,10].

First-line therapies for mild and moderate acne comprise topical agents with keratolytic properties—such as benzoyl peroxide, salicylic acid, and retinoids—as well as topical or systemic antibiotics. More severe or persistent disease forms may necessitate oral antibiotics, hormonal agents (notably in women presenting with endocrine abnormalities), or oral isotretinoin. Despite their effectiveness, antibiotic overuse has become a significant public health issue, contributing to the rise in antibiotic-resistant *C. acnes* strains and long-term perturbations of the skin and gut microbiomes. This underscores the necessity for alternative, targeted interventions and continued research into safe, sustainable acne management approaches [12,14,17,18,19].

According to the latest guidelines from the European Academy of Dermatology and Venereology (JEADV), oral antibiotics are no longer recommended as a first-line treatment for acne. Their use should be limited and never as monotherapy due to growing concerns about antibiotic resistance. When oral antibiotics are deemed necessary for moderate to severe acne, they should be combined with topical benzoyl peroxide to reduce resistance risk and used for the shortest effective duration, typically fewer than 3–4 months. The guidelines instead prioritize non-antibiotic therapies such as topical retinoids, benzoyl peroxide, and hormonal treatments as preferred first-line options [20]. Despite these recommendations, antibiotic therapy remains relatively common in practice, which underscores the importance of antibiotic stewardship to minimize the risk of developing antibiotic-resistant strains of *C. acnes* and to ensure the long-term effectiveness of acne treatments. As antibiotic resistance among skin flora becomes more prevalent and the chronic, relapsing nature of acne vulgaris challenges both patients and clinicians, there is a growing emphasis on supplementary and alternative management strategies [18]. Alongside established pharmacological approaches, supportive treatments such as chemical skin stimulants, phototherapy, physical, chemical or mechanical exfoliation, and specialized procedures are frequently explored to enhance outcomes, particularly for patients with suboptimal responses to classic regimens [21,22,23].

Chemical peels stand out as one of the most widely adopted methods to support acne care. Both superficial peels—formulated with agents such as mandelic, pyruvic, or salicylic acids—and medium-depth treatments using mixtures, like Jessner’s solution, yellow peel, or trichloroacetic acid (TCA), are applied to improve skin clarity, reduce inflammation, and minimize post-inflammatory hyperpigmentation in diverse patient populations.

Nofal et al. [22] demonstrated that combining modified Jessner’s solution with 20% TCA resulted in significantly greater improvement in acne-prone skin parameters than using peels alone. The inflammatory lesion count in the combination peel group decreased significantly from 27.2 ± 5.3 lesions at baseline to 7.6 ± 2.7 post-treatment, whereas the single peel group exhibited a reduction from 26.8 ± 5.8 to 13.4 ± 3.1 lesions (*p* < 0.05). Non-inflammatory lesions (comedones) in the combination peel group decreased from 34.5 ± 6.1 to 11.2 ± 3.4 lesions, outperforming the single-peel group’s improvement from 35.1 ± 5.9 to 19.7 ± 4.3 lesions (*p* < 0.05). Additionally, sebum secretion was reduced by 38% in the combination peel group compared to 21% in the single-peel group, as measured by sebumetry (*p* < 0.05). The results of the study by Jae et al. [24] indicate that chemical peeling using a 50% glycolic acid (GA) solution (pH 3.0) + 0.5% salicylic acid (SA) solution may be as effective and convenient as conventional peeling using Jessner’s solution in the treatment of acne vulgaris, and may also cause fewer adverse effects than conventional peeling. Glycolic acid, as the smallest α-hydroxy acid, facilitates controlled keratolysis and cellular renewal by decreasing intercellular cohesion while enhancing hydration and stimulating collagen synthesis. Studies have confirmed that glycolic acid peels (35–70%) effectively reduce comedonal acne and atrophic scars through epidermal remodeling and seboregulation [25,26]. Salicylic acid, a β-hydroxy acid, penetrates sebaceous follicles due to its lipophilic nature, exhibiting potent comedolytic, sebostatic, and anti-inflammatory activity primarily through the inhibition of cyclooxygenase (COX) enzymes, which reduces the synthesis of pro-inflammatory prostaglandins. Additionally, salicylic acid modulates nuclear factor kappa B (NF-κB) signaling, a key transcription factor involved in inflammatory responses, thereby decreasing the production of inflammatory cytokines such as tumor necrosis factor-alpha (TNF-α) and interleukins (IL-1, IL-6) leading to reduced sebum secretion and inflammation in acne lesions. Salicylic acid effectively suppresses sebum production and reduces inflammation in human sebocytes (SEB-1 cells) by inhibiting the AMPK/SREBP-1 signaling pathway. Specifically, salicylic acid activates AMP-activated protein kinase (AMPK), a key energy-sensing enzyme that negatively regulates lipid synthesis. Activation of AMPK subsequently downregulates sterol regulatory element-binding protein 1 (SREBP-1), a transcription factor critical for fatty acid and lipid biosynthesis in sebocytes. This suppression leads to decreased lipid production, which is beneficial in acne treatment by reducing the oily environment that promotes acne lesion formation. Its keratolytic property also helps by exfoliating the stratum corneum and opening clogged pores, reducing the inflammatory stimulus associated with follicular occlusion [27,28,29,30]. Azelaic acid provides antibacterial and anti-inflammatory effects by inhibiting *C. acnes* growth and modulating keratinocyte differentiation. It also acts as an inhibitor of 5 α reductase and tyrosinase, which decreases sebum production and post-inflammatory hyperpigmentation [31,32,33]. Clinical studies have shown that azelaic acid peels (20–30%) improve both inflammatory and non-inflammatory acne while evening out skin tone [33,34]. Kojic acid and phytic acid contribute potent depigmenting effects through tyrosinase inhibition and metal chelation, reducing melanin synthesis and phototoxic pigmentation [35]. The retinaldehyde (vitamin A) component enhances epidermal turnover, normalizes keratinization, and promotes neocollagenesis by activating nuclear receptors RAR (Retinoic Acid Receptor) and RXR (Retinoid X Receptor). Retinoids are proven to improve scar depth, elasticity, and pigmentation by upregulating collagen I and III synthesis and inhibiting matrix metalloproteinases [36]. Together, these ingredients produce a broad-spectrum dermatologic effect, comprising exfoliation and remodeling of the epidermis, stimulation of dermal regeneration, suppression of inflammation and seborrhea, and reduction in dyspigmentation.

The aim of this study was to quantitatively evaluate the effectiveness of a medium-depth chemical peeling protocol (yellow peel) in alleviating the symptoms of acne vulgaris in a precisely defined population of young adults aged 20–25 years. In designing the trial, particular emphasis was placed on this age group because early intervention at a mild-to-moderate stage of disease may substantially modify the clinical trajectory and reduce the long-term burden of atrophic scarring. To obtain robust and clinically meaningful data, the study incorporated both conventional clinical grading (including IGA and inflammatory lesion counts) and advanced 3D imaging to objectively quantify scar depth, alongside measurements of skin hydration and sebum production at repeated time points.

## 2. Materials and Methods

### 2.1. Study Participants

The study included 20 subjects (17 women and 3 men; phototype II) aged 20 to 25 who suffered from light or moderate acne lesions located on the face. The classification of acne severity was based on the Investigator’s Global Assessment (IGA) scale. Qualifications for treatments were performed by a dermatologist based on a protocol and taking into account the adopted inclusion and exclusion criteria. The inclusion criteria were as follows: patients over 18 years of age who could decide on their own about participation in the study, patients with inflammatory lesions and atrophic scars in the course of acne vulgaris eligible for chemical peeling therapy, no general or local antibiotic therapy in the area assessed, no treatment with general and local steroid drugs in the area assessed, male and female patients, patients able to understand the purpose and risks associated with the study after signing the patient informed consent form to participate in the study. The exclusion criteria included pregnancy or breastfeeding, bacterial skin diseases other than acne vulgaris, viral, parasitic, fungal infections (especially tuberculosis), HIV infection, HBV infection, people diagnosed with systemic lupus erythematosus, demyelinating diseases, active cancer and severe circulatory failure, lack of informed consent and cooperation of the patient, and abuse of drugs or alcohol by the patient revealed in the interview.

### 2.2. Procedure of Chemical Peeling

Twenty patients, selected by a dermatologist for the study, underwent two sessions of treatment with a medium-depth chemical peel—yellow peel—administered at four-week intervals. Prior to each treatment, the skin was cleansed with a pH 5.5 cleansing agent and then gently dried. The first step of the peeling procedure involved the application of 20% glycolic acid, pH 3.7 (pre-peeling cleanser, Mene & Moy, Dermatologic Skin Care Solutions LLC, Doral, FL, USA; ICNI: water (aqua), glycolic acid, TEA-lauryl sulfate, ammonium hydroxide, sodium lauryl sulfate, propylene glycol, glycol distearate, hydroxethylcellulose, parfum, tetradosium EDTA, alpha-isomethyl ionone, sodium hydroxide, geraniol, amyl cinnamal) to the skin, which was left on for five minutes before being rinsed off with cold water and patted dry with a paper towel. The second stage consisted of applying a mixture containing 40% glycolic acid and 10% salicylic acid, pH 1.0 (alpha–beta complex gel, Mene & Moy, Dermatologic Skin Care Solutions LLC, Doral, FL, USA; INCI: glycolic acid, SD alcohol 40, water, propylene glycol, salicylic acid ammonium hydroxide, hydroxyethylcellulose, tetrasodium EDTA) over the entire face using a brush. The duration of exposure varied individually but generally lasted from one to three minutes, with removal timed to the onset of mild erythema across the face. The peel was then rinsed off with cold water, and the skin was dried with a paper towel. In the third phase, a mask composed of retinol, azelaic acid, phytic acid, kojic acid, salicylic acid (yellow peel, Mene & Moy, Dermatologic Skin Care Solutions LLC, Doral, FL, USA; INCI: Laneth 16, lanolin, petrolatum, retinol, azelaic acid, phytic acid, koic acid, emu oil, salicylic acid) was applied. After gentle massage into the skin, a second layer of the mask was added. This yellow mask was left on the skin for four hours, after which it was removed using water and a gentle cleanser formulated for sensitive skin. Following drying, a moisturizing cream containing hyaluronic acid and a broad-spectrum sunscreen with SPF 50+ were applied. Participants were advised to use only the Avene Cicalfate healing cream (INCI: Avene Thermal Spring Water (Avene Aqua), caprylic/capric triglyceride, mineral oil (Paraffinum Liquidum), glycerin, hydrogenated vegetable oil, zinc oxide, propylene glycol, polyglyceryl-2 sesquiisostearate, Peg-22/dodecyl glycol copolymer, aluminum stearate, Aquaphilus Dolomiae ferment filtrate, arginine, beeswax (Cera Alba), copper sulfate, magnesium stearate, magnesium sulfate, microcrystalline wax (Cera Microcristallina), tromethamine, zinc sulfate), SPF 50+ sunscreen and mild cleansers for four weeks post-treatment.

### 2.3. Photographic Documentation and Biomechanical Measurements

Before the first treatment, 28 days after the first treatment and 2 months after the second treatment, photographic documentation, 3D images and biomechanical measurements of the patients’ skin were performed.

To prepare photographic documentation, the Fotomedicus clinical photography kit from Elfo (Dębica, Poland), which allows for image acquisition in unpolarized and cross-polarized light, was used. The number of inflammatory lesions on the left and right sides of the face was counted using clinical photographs. The severity of acne was assessed using the widely accepted Investigator’s Global Assessment (IGA) scale. The IGA is a five-point scale ranging from 0 to 4, where 0 indicates clear skin with no lesions; 1 corresponds to almost clear skin with minimal lesions and slight symptoms; 2 denotes mild severity with noticeable but mild lesions; 3 reflects moderate severity with marked lesions present across several facial areas; and 4 signifies severe acne with numerous extensive lesions, significant inflammation, scarring, and cysts. This assessment considers not only the quantity of lesions, but also their types—including comedones, papules, pustules, nodules, and scars—as well as their distribution and extent.

Three-dimensional images of the skin surface, along with measurements of 6 skin parameters, were acquired using the Antera 3D device from Miravex Limited (Dublin, Ireland). The scar depth was assessed by analyzing images acquired with the Antera 3D device. In each image, the area with the largest total volume of scars was selected on both the right and left cheeks.

Skin hydration and sebum level were measured using a corneometer and sebumeter from Courage-Khazaka Electronic (Köln, Germany). Measurements of skin hydration and sebum level were made in the entire area of skin irradiation, i.e., in the middle of the forehead between the base of the eyebrows, on the tip of the nose, in the middle of the chin, and on the highest points of the zygomatic bone on both the right and left sides of the face. Both stratum corneum hydration and sebum level were measured in arbitrary units (a.u.).

**Figure 1:**
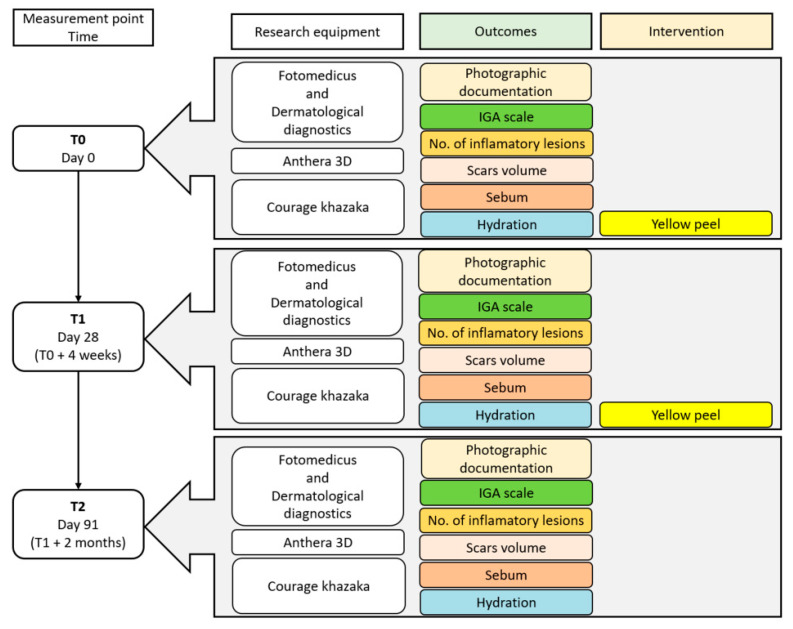
A schematic timeline of the treatment schedule and follow-up visits.

A schematic timeline of the treatment schedule and follow-up visits is presented in Figure 1.

The research was carried out after obtaining the written consent of volunteers who were familiarized with the purpose of the research and its course, and with the consent of the Ethics Committee of the Medical University of Silesia No. PCN/0022/KB1/11/I/20 on 19 May 2020. The research was conducted from October 2024 to April 2025.

### 2.4. Statistical Analysis

After performing the measurements, all results were placed in Excel sheets, and then exported to Statistica 13.3, where statistical analysis and figures were performed. The impact of the medium-depth peel on acne skin condition, evaluated using the Investigator’s Global Assessment (IGA) scale, was analyzed with the Chi-squared test. Subsequently, the normality of the distribution for all measurements—including the number of inflammatory lesions, scar volume, sebum levels, and skin hydration—was assessed using histograms and the Shapiro–Wilk test. As a substantial proportion of the data did not meet the normality criteria, statistical analysis of these variables was conducted using the Friedman ANOVA test accompanied by Dunn’s post hoc multiple comparisons. Results were considered statistically significant at a *p*-value less than 0.05 [37,38].

## 3. Results

**Figure 2:**
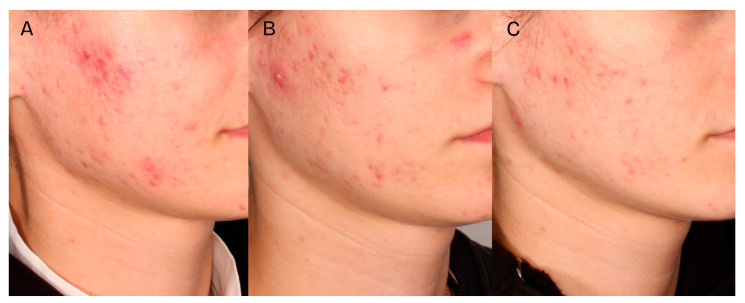
Patient 1, skin condition before the first treatment—(**A**), 4 weeks after the first treatment—(**B**), 2 months after the second treatment—(**C**).

**Figure 3:**
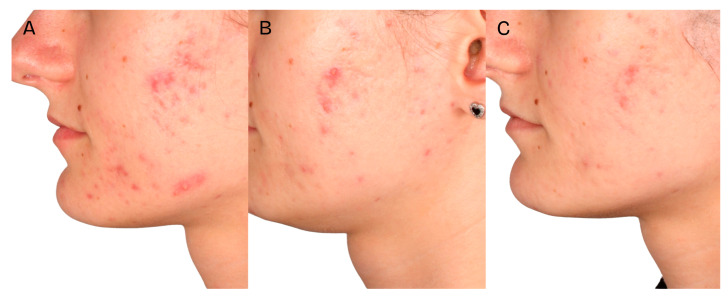
Patient 2, skin condition before the first treatment—(**A**), 4 weeks after the first treatment—(**B**), 2 months after the second treatment—(**C**).

**Figure 4:**
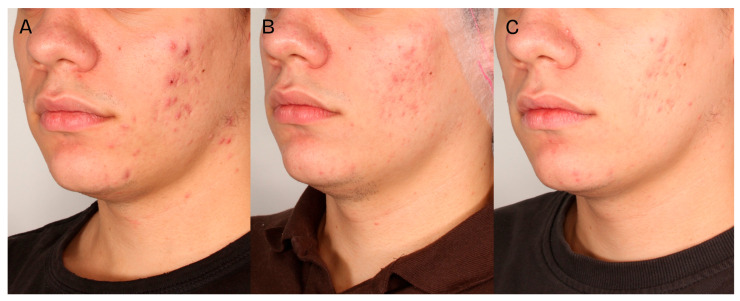
Patient 3, skin condition before the first treatment—(**A**), 4 weeks after the first treatment—(**B**), 2 months after the second treatment—(**C**).

**Figure 5:**
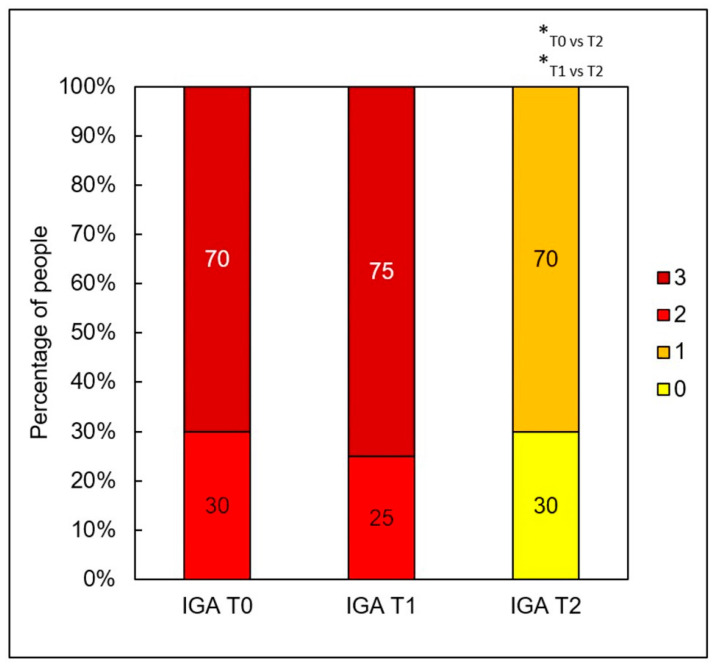
Percentage of subjects by IGA score before treatment (T0), four weeks after one treatment (T1), and two months after two treatments (T2); * *p* < 0.001.

A significant clinical improvement in the condition of the volunteers’ skin was observed after a series of two medium-depth chemical peel treatments (Figure 2, Figure 3 and Figure 4). The procedures produced a notable reduction in acne severity according to the IGA scale (*p* < 0.001) (Figure 5). Post hoc analysis indicated that statistically significant differences were observed between the IGA values recorded before treatment (T0) and two months after completing both treatments (T2) (*p* < 0.001), as well as between the measurements taken four weeks after the first peel (T1) and two months after the second peel (T2) (*p* < 0.001). At T0 and T1, most patients exhibited an IGA score of 3, whereas after two months following the second peel, 70% of participants achieved an IGA score of 1 and 30% reached an IGA score of 0.

**Figure 6:**
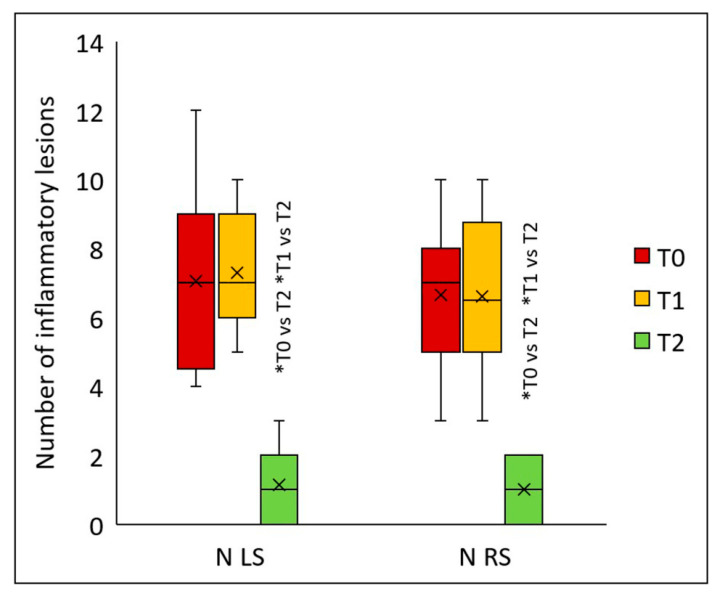
Number of inflammatory lesions in a group of 20 patients on the left (LS) and right side (RS) of the face before treatment (T0), four weeks after one treatment (T1), and two months after two treatments (T2); * *p* < 0.001.

Similarly, the peels significantly reduced the number of inflammatory lesions on both the left (*p* < 0.001) and right sides of the face (*p* < 0.001) (Figure 6). Statistically significant differences for both facial sides were detected two months after the final peel (T2) versus values at T1 (*p* < 0.001) and T0 (*p* < 0.001). On the left side, the median number of inflammatory lesions was 7 (T0), 7 (T1), and 1 (T2); on the right side, 7 (T0), 6.5 (T1), and 1 (T2). Compared to baseline, two months after the second peel, the count of inflammatory lesions declined by 85.7% on both facial sides.

**Figure 7:**
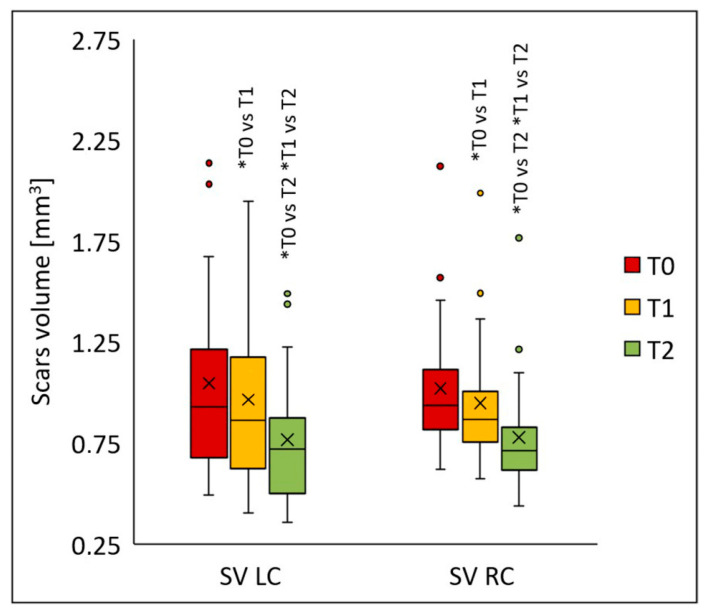
Scar volume (SV) on the left (LC) and right cheek (RC) before treatment (T0), four weeks after one treatment (T1), and two months after two treatments (T2); * *p* < 0.001.

The procedures also led to a significant reduction in scar volume on both the left (*p* < 0.001) and right cheeks (*p* < 0.001) (Figure 7). Statistically significant differences for both cheeks appeared following the first peel (T1) (*p* < 0.005), and the values decreased even further two months after the final peel (T2) compared to baseline (T0) (*p* < 0.001). On the left cheek, the median scar volume was 0.926 (T0), 0.863 (T1), and 0.718 (T2); on the right cheek, 0.938 (T0), 0.868 (T1), and 0.713 (T2). Compared to baseline, the median scar volume declined by 6.9% (T1) and 22.5% (T2) on the left cheek, and by 7.5% (T1) and 23.9% (T2) on the right cheek.

**Figure 8:**
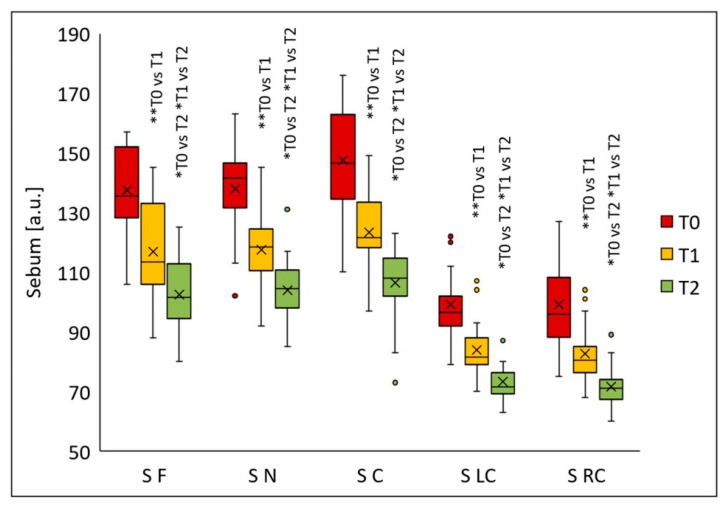
Sebum secretion (S) on the forehead (F), nose (N), chin (C), left cheek (LC), and right cheek (RC) before treatment (T0), four weeks after one treatment (T1), and two months after two treatments (T2); * *p* < 0.001, ** *p* = 0.005.

Sebum secretion decreased significantly in all analyzed facial regions following the chemical peel procedures (Figure 8). Significant differences were noted in all evaluated areas after the first peel (T1) (*p* < 0.005); further sebum reduction was observed two months post-final peel (T2) compared with the values at T0 (*p* < 0.001). The median sebum values were as follows: forehead—135.5 (T0), 113.5 (T1), 101.5 (T2); nose—141.5 (T0), 118.5 (T1), 104.5 (T2); chin—146.5 (T0), 121.5 (T1), 108.0 (T2); left cheek—96.5 (T0), 81.5 (T1), 71.5 (T2); right cheek—96.0 (T0), 80.5 (T1), 71.0 (T2). Relative to baseline, median sebum secretion decreased 16.2% (T1) and 25.1% (T2) on the forehead, 16.3% (T1) and 26.1% (T2) on the nose, 17.1% (T1) and 26.3% (T2) on the chin, 15.5% (T1) and 25.9% (T2) on the left cheek, and 16.1% (T1) and 26.0% (T2) on the right cheek.

**Figure 9:**
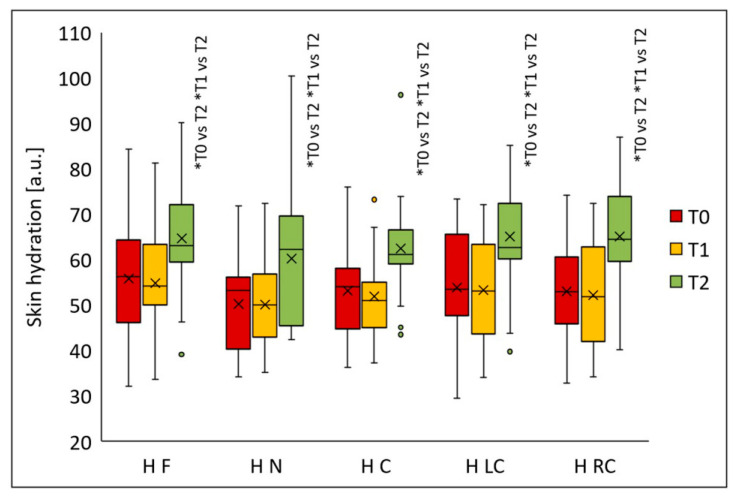
Skin hydration (H) on the forehead (F), nose (N), chin (C), left cheek (LC), and right cheek (RC) before treatment (T0), four weeks after one treatment (T1), and two months after two treatments (T2); * *p* < 0.001.

Skin hydration increased significantly in all analyzed facial regions after the peeling procedures (*p* < 0.001) (Figure 9). Statistically significant differences in all areas were observed two months after the final peel (T2) versus T1 (*p* < 0.001) and T0 (*p* < 0.001). Median hydration values were: forehead—56.2 (T0), 54.2 (T1), 63.0 (T2); nose—53.3 (T0), 50.0 (T1), 62.2 (T2); chin—54.0 (T0), 50.9 (T1), 61.1 (T2); left cheek—53.5 (T0), 53.0 (T1), 62.7 (T2); and right cheek—53.0 (T0), 51.8 (T1), 64.4 (T2). Compared to baseline, median skin hydration increased after two months from the last treatment by 12.1% on the forehead, 16.7% on the nose, 13.1% on the chin, 17.2% on the left cheek, and 21.6% on the right cheek.

It is worth noting that adverse reactions were typical of medium-depth chemical peels, including transient skin redness and moderate peeling lasting approximately 4–5 days. No serious adverse reactions were observed.

## 4. Discussion

The present study quantitatively evaluated the effectiveness of a medium-depth yellow peel protocol in alleviating the symptoms of acne vulgaris in a narrowly defined cohort of young adults aged 20–25 years. This age group was deliberately selected because mild-to-moderate acne at this stage often coincides with the early development of atrophic scars, and timely intervention may modify the subsequent clinical course and reduce the long-term scarring burden. By focusing on this specific demographic, the study provides clinically relevant data for a population that is highly affected by acne yet relatively underrepresented in trials of medium-depth peels. The multimodal yellow peel regimen used in this trial combined glycolic acid, salicylic acid, and a complex mask containing retinol, azelaic, phytic, kojic, and salicylic acids, thereby targeting several key pathogenic mechanisms of acne, including abnormal keratinization, seborrhea, *Cutibacterium acnes*–driven inflammation, and post-inflammatory hyperpigmentation. The significant reductions in inflammatory lesion counts and objective scar depth, together with improved skin hydration and reduced sebum secretion, support the hypothesis that such a layered approach can provide broader therapeutic benefits than single-agent peels reported in earlier work. These findings are in line with previous studies on combination chemical peels, but extend existing evidence by demonstrating comparable efficacy with a different, clinically available formulation applied as a medium-depth protocol in young adults. These findings broadly align with the recent scientific literature analyzing the effects of chemical peels containing glycolic acid, salicylic acid, azelaic acid, retinoic acid, and medium-depth peels such as TCA, and they extend current evidence through the use of multimodal measurements and contemporary imaging techniques [38]. Randomized clinical trials have demonstrated that glycolic acid peels (typically 40–70%) lead to significant reductions in both inflammatory and non-inflammatory acne lesions [39]. Kaminaka et al. [28] found that a 40% glycolic acid peel administered every two weeks resulted in a statistically significant improvement in moderate acne cases, including reductions in lesion counts and sebum levels, with excellent safety in Asian skin types. Comparative studies show that salicylic acid peels (especially 30%) are equally effective, and may have a more pronounced impact on reducing oiliness and comedonal lesions. In systematic reviews, both glycolic and salicylic acid were confirmed as safe, effective options for mild-to-moderate acne vulgaris, with similar reductions in IGA scores and patient satisfaction rates [39,40]. Several studies highlight that combining acids or alternating protocols can amplify treatment outcomes. For example, Nofal et al. [21] reported that modified Jessner’s solution (with lactic, salicylic, and citric acids) plus TCA resulted in greater improvement in acne parameters than single peels. Jae et al. [27] compared 50% glycolic acid plus 0.5% salicylic acid with conventional Jessner’s solution, noting comparable efficacy but fewer adverse reactions. Azelaic acid peels are increasingly recognized for their dual anti-inflammatory and depigmenting properties [34]. Chilicka et al. [41] found that six sessions of azelaic acid in 120 young women with papulopustular acne led to significant reductions in severity scores, oiliness, and desquamation, with results comparable to pyruvic acid peels. Recent meta-analyses suggest that the inclusion of azelaic acid in chemical peels enhances both antibacterial activity and restoration of skin barrier function [34]. Retinoic acid peels show particularly robust efficacy in decreasing inflammatory acne lesions and improving IGA endpoints. Bassant Anwar et al. [42] confirmed that a 5% retinoic acid peel not only reduced acne scores more than 30% salicylic acid but also resulted in higher patient satisfaction. Additional studies demonstrate that retinoic acid promotes cell renewal and dermal remodeling, leading to superior exfoliation and better improvement of atrophic scarring than glycolic acid alone.

Medium-depth peels, especially TCA at 30%, are well-established in the management of atrophic acne scars. Manjhi et al. [43] found TCA peels to elicit faster and more pronounced improvements in scar depth compared to high-strength glycolic acid (70%), although postinflammatory hyperpigmentation and dryness were observed more often with TCA. Both treatments produced significant reductions in mean scar volume, acne severity, sebum secretion, and improvements in patient-rated esthetic outcomes. In the cited study, the skin was prepared for the peel with a 0.025% tretinoin cream applied for 2 weeks. A reduction in scar depth of over 20% was achieved on the side treated with 30% TCA peels. In our study, we obtained a similar reduction in scar depth using a yellow peel mixture.

Most clinical trials and quantitative studies concur that chemical peels reliably improve skin hydration and reduce excessive sebum secretion—a key factor in acne pathogenesis. Odrzywołek et al. [44] noted improvements in skin texture following chemical peels as measured by GLCM analysis and expert panel ratings, with significant changes in hydration and inflammation parameters. These effects are observed with glycolic acid, salicylic acid, and azelaic acid, but multimodal protocols including retinoic acid appear to offer additional benefit for restoring barrier function and moisture.

The reductions in IGA scores, number of inflammatory lesions, scar volume, sebum production, and increases in skin hydration documented in this study mirror findings reported in randomized trials and meta-analyses of chemical peeling roles in acne management. The multimodal yellow peel protocol incorporating glycolic, salicylic, retinol, azelaic, and other acids is consistent with recent recommendations that combination peels yield greater clinical benefit and patient satisfaction, with acceptable safety profiles. Overall, the evidence from the past decade confirms chemical peels—including glycolic, salicylic, azelaic, retinoic acid, and TCA—as effective interventions in improving clinical severity (IGA), scar depth, skin hydration, sebum regulation, and reduction in active acne lesions, supporting their continued and expanded use in dermatological practice for acne vulgaris. The sustained improvements observed two months after the second peel indicate not only short-term cosmetic benefit but also a potential remodeling effect on atrophic scars and skin barrier function. This multi-parameter strategy offers a more nuanced understanding of how medium-depth peels influence both clinical and subclinical features of acne-prone skin.

### Study Limitations

This study has several limitations that warrant consideration. First, the sample size was relatively small, limiting the generalizability of the findings and the statistical power to detect less pronounced effects or rare adverse events. Second, the narrow age range of 20–25 years further limits the generalizability of our findings to older patients, adolescents, or individuals with severe forms of acne. Third, the study design did not include a placebo or alternative treatment control group, which limits the ability to firmly attribute clinical improvements solely to the yellow peel regimen. Fourthly, the follow-up period was relatively short, restricting insights into the long-term efficacy and safety of the peel protocol, especially regarding scar remodeling sustainability and potential late adverse effects. Fifthly, home skincare regimens were not fully standardized, and individual variation in adherence to post-peel instructions may have introduced unmeasured variability into the results. Additionally, the study population was restricted to patients with mild to moderate acne vulgaris and limited geographic and ethnic diversity, which may affect the applicability of the results to broader or more diverse patient groups. Lastly, subjective assessments, while supported by objective measures such as 3D scar depth imaging, could introduce some observer bias despite blinding efforts.

Future studies with larger, multi-center cohorts, longer follow-up, and randomized controlled designs including comparator arms will be necessary to fully establish the therapeutic value and safety profile of the yellow peel in diverse clinical settings.

## 5. Conclusions

The multimodal medium-depth yellow peel protocol produced consistent clinical and instrumental improvements in young adults with mild-to-moderate acne and early atrophic scars, including reductions in inflammatory lesion counts and scar depth, together with increased skin hydration and reduced sebum secretion. Although the study was an exploratory, single-center pilot with a small, age-restricted sample and non-standardized home skincare, the use of appropriate non-parametric statistics and multiple complementary outcome measures supports the robustness of the observed trends. These findings suggest that sequential yellow peel sessions may be a valuable adjunctive option in the early, targeted management of acne vulgaris in young adults, but larger, randomized controlled trials with longer follow-up are required to confirm efficacy and generalizability.

## Data Availability

Dataset available on request from the authors.
